# Analysis of the Metabolic Response of Planktonic Cells and Biofilms of *Klebsiella pneumoniae* to Sublethal Disinfection with Sodium Hypochlorite Measured by NMR

**DOI:** 10.3390/microorganisms10071323

**Published:** 2022-06-30

**Authors:** David Felipe Garcia Mendez, Julián Andrés Rengifo Herrera, Janeth Sanabria, Julien Wist

**Affiliations:** 1 Chemistry Department, Universidad del Valle—Sede Meléndez, Cali 13 # 100-00, Colombia; david.garcia.mendez@correounivalle.edu.co (D.F.G.M.); julien.wist@murdoch.edu.au (J.W.); 2 Australian National Phenome Center, Murdoch University, Perth, WA 6150, Australia; 3 Centro de Investigación y Desarrollo en Ciencias Aplicadas “Dr. J.J. Ronco” (CINDECA), Departamento de Química, Facultad de Ciencias Exactas, UNLP-CCT La Plata, CONICET, 47 No. 257, La Plata 1900, Argentina; julianregifo@quimica.unlp.edu.ar; 4 Environmental Microbiology and Biotechnology Laboratory, Engineering Faculty, Engineering School of Environmental & Natural Resources, Universidad del Valle—Meléndez Campus, Cali 13 # 100-00, Colombia

**Keywords:** exometabolome, metabolism, metabolite, chlorine, chlorination, NaOCl, Klebsiella, water

## Abstract

*Klebsiella pneumoniae* is a pathogenic agent able to form biofilms on water storage tanks and pipe walls. This opportunistic pathogen can generate a thick layer as one of its essential virulence factors, enabling the bacteria to survive disinfection processes and thus develop drug resistance. Understanding the metabolic differences between biofilm and planktonic cells of the *K. pneumoniae* response to NaClO is key to developing strategies to control its spread. In this study, we performed an NMR metabolic profile analysis to compare the response to a sublethal concentration of sodium hypochlorite of biofilm and planktonic cells of *K. pneumoniae* cultured inside silicone tubing. Metabolic profiles revealed changes in the metabolism of planktonic cells after a contact time of 10 min with 7 mg L^−1^ of sodium hypochlorite. A decrease in the production of metabolites such as lactate, acetate, ethanol, and succinate in this cell type was observed, thus indicating a disruption of glucose intake. In contrast, the biofilms displayed a high metabolic heterogeneity, and the treatment did not affect their metabolic signature.

## 1. Introduction

According to the WHO and UNICEF (2021) [1], 771 million people do not have access to safe drinking water, including 282 million who rely on “limited” water services (improved sources where water collection takes longer than 30 min), 367 million who relied on improper sources, and 122 million who drank directly from rivers, lakes, and other surface water sources. Biofilms in wells, household storage, and drinking water distribution systems (DWDSs) can be a potential reservoir for pathogens. They can gain protection against disinfectants and develop resistance to antibiotics [2]. The release of microorganisms may be caused by seasonal changes or operative adjustments, triggering infection outbreaks [3]. Additionally, biofilms may alter the taste and odor of drinking water and corrode the pipes [4].

The formation of biofilms is a natural survival strategy for microorganisms. They attach to a surface and are enclosed by themselves within an extracellular matrix. In DWDSs, their growth is favored by the pipe material [5], residues of organic matter present in water, and poor disinfection systems [6]. On the other hand, it is calculated that up to 80% of all human body infections are biofilm-related. Biofilms can colonize different medical devices and cause chronic infections, increasing mortality and morbidity rates. They also show an outstanding antimicrobial resistance up to 1000-fold compared to their planktonic counterparts [7].

Different factors promote or inhibit the growth of biofilms. It is well known that biofilm formation occurs in different phases involving reversible and irreversible attachment, maturation, and dispersion. The development of biofilm at a surface results from physical, chemical, and microbial processes, including pH, hydrodynamics, osmolarity, presence of specific ions, nutrients, and factors derived from the biotic environment [8]. The transition from the planktonic to the biofilm state involves the interaction of several proteins and regulatory systems, such as 3′,5′-cyclic diguanylic acid (c-di-GMP), two-component signaling systems (TCS), the RcsCDB regulator, and quorum sensing (QS) [9]. Other factors contributing to *K. pneumoniae* biofilm formation include, among others, the presence of a polysaccharide capsule, lipopolysaccharide (LPS), fimbriae, pili, iron metabolism, and the presence of other bacterial species [10,11].

Once formed, biofilms exhibit outstanding resistance to harsh conditions. They can survive the presence of disinfectants, shear forces, thermal stress, and predators [4]. The resistance mechanisms of biofilms include the physical barrier provided by the extracellular matrix, which decreases antimicrobial penetration; alteration of the outer membrane; the production of enzymes that transform biocides into non-toxic forms; persister cells; efflux pumps; high heterogeneity in metabolism and growth rates; horizontal gene transfer [9,12]; and adaptive mutagenesis [13].

The stress response is also markedly different in planktonic and biofilm cells. The study of biofilm models such as *P. aeruginosa* [14,15], *P. fluorescens* [16,17], *S. aureus* [18,19] and *E. coli* [20] have revealed the complex intracellular and extracellular regulatory pathways required for the coordination and control of the biofilm response. This coordinated cell-to-cell communication allows the biofilm to change motility and adherence, their energy metabolism, physiology, and the production of molecules that are constituents of the extracellular matrix to increase their survivability. These features cause a sharp increase in the disinfectant concentration and contact times required for their elimination, with negative consequences for drinking water quality.

To the best of our knowledge, this is the first study that compares the metabolic response of planktonic and biofilm cells of *K. pneumoniae* to chlorination. This pathogen is frequently found in healthcare-associated infections, and biofilm formation is one of the resistance mechanisms that allow its persistence after water chlorination and being found inside DWDS [4] and storage wells of groundwater [21]. A better understanding of the fundamental metabolic pathways that allow the persistence and formation of biofilms are needed. It can improve the development of strategies for preventing their formation in environments where they can be harmful. NMR is a robust technique that has been successfully used to accurately identify and quantify critical metabolites, providing valuable insights into biofilm metabolism [22]. The main aim of this study was to investigate the differences between the NMR metabolic profiles of planktonic cells and biofilms of *K. pneumoniae* exposed to sublethal concentrations of sodium hypochlorite.

## 2. Materials and Methods

### 2.1. Bacterial Strain Reactivation

All the experiments were performed using the strain ATCC 13882 of *K. pneumoniae*. For its reactivation, the cryopreserved strain was grown at 37 °C in CASO agar for 18 h. One bacterial colony was transferred to 40 mL CASO broth and was incubated at 37 °C with constant stirring for 18 h when the steady-state was reached. The cells were harvested from the total volume by centrifugation for 10 min at 3240 RCF and discarding the supernatant. The pellet was washed twice with 1× PBS and resuspended to its original volume.

### 2.2. Culture of Biofilm and Planktonic Cells

**Figure 1:**
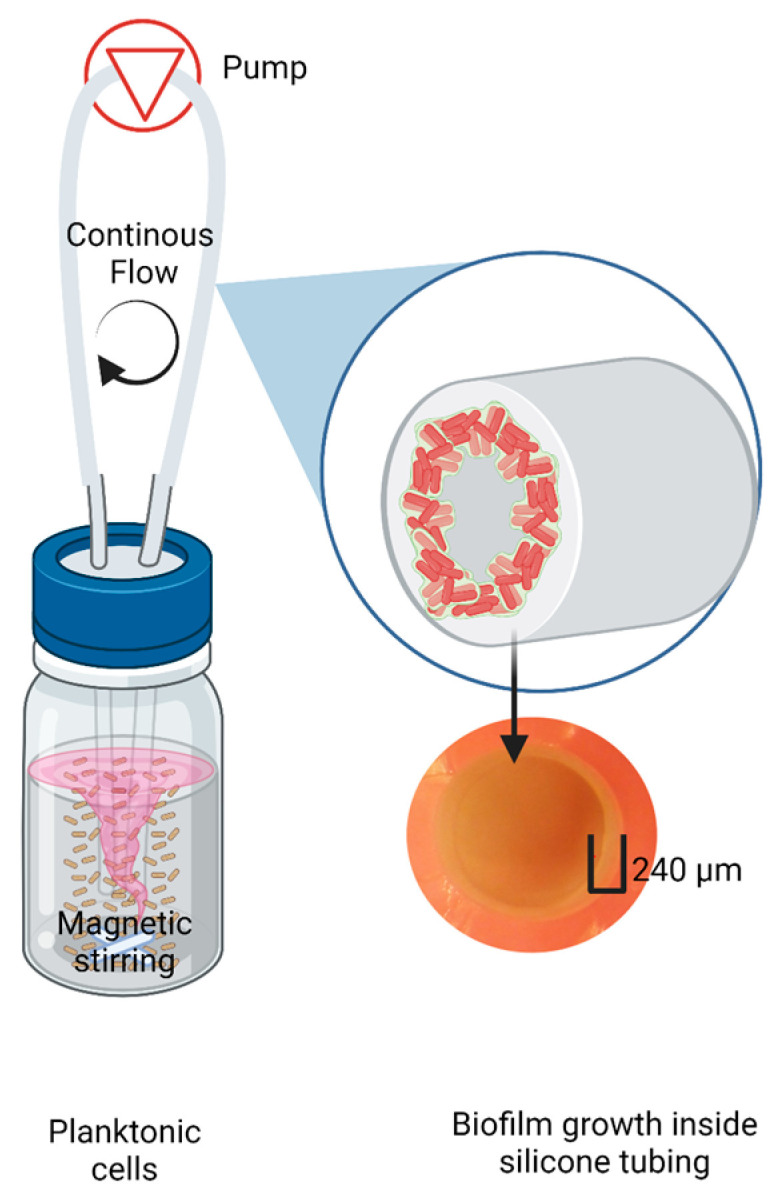
Scheme of experimental units for disinfection treatment of biofilms and planktonic cells of *K. pneumoniae*. Each treatment and control had three replicates. Image created with BioRender.com.

Biofilms of *K. pneumoniae* were obtained by continuously circulating a bacterial culture through a food-grade silicone tube (OD = 3.8 mm, ID = 2.8 mm) using a peristaltic pump at 0.5 mL min^−1^, as shown in Figure 1. The complete culture media was replaced seven times every 18 h to maintain the remaining cells at their maximum growth rate. The cells that grew in the bottle in the last cycle (as planktonic cells) were harvested for the following steps. The bacterial culture was initiated in 100 mL of nutrient media (casein peptone: 8.5 gL^−1^, dipotassium hydrogen phosphate: 1.25 gL^−1^, D(+)-glucose: 1.25 gL^−1^, sodium chloride: 2.5 gL^−1^, soy peptone: 1.5 gL^−1^) in a 1 L bottle. The media was inoculated with a bacterial suspension to an OD_600_ = 0.1 (equivalent to 3 × 10^8^ bacteria mL^−1^) and incubated at 37 °C with magnetic stirring at 300 rpm. Six replicates were run.

The bottles with planktonic cells and the biofilms in the silicone tubes were separated. The bacteria were washed before disinfection. In the case of biofilms, the silicone tubes were connected to a bottle with 100 mL of a sterile saline solution (NaCl 0.85%), which was recirculated for 10 min. The wash process was repeated for the planktonic cells, as described previously for bacterial strain reactivation.

### 2.3. Minimum Inhibitory Concentration of the Disinfectant in Planktonic Cells

In total, 200 mL of CASO broth were inoculated to an initial OD_600_ = 0.1 (equivalent to 3 × 10^8^ bacteria mL^−1^) and incubated at 37 °C for 6 h. Samples of 28 mL were transferred to a Falcon tube and washed, as described previously. The bacterial cells were resuspended in saline solution (NaCl 0.85%) to an OD_600_ = 2.5.

The minimum inhibitory concentration of sodium hypochlorite was determined using the plate microdilution method, with some modifications. In a 96-well microtiter plate, 100 μL of distilled water was added to each plate. In total, 100 μL of a solution of 245 mg L^−1^ of sodium hypochlorite were added to the first well. From this well, serial dilutions of 1:2 were performed until reaching a final concentration of 1.9 mgL^−1^. Then, 100 μL of the bacterial suspension obtained previously was added to each well. The plate was incubated at 37 °C for 40 min, and 25 μL from each well was transferred to a new microtiter plate that contained 175 μL of CASO broth. The OD_600_ of each well was measured, and OD_600_ > 0.1 was considered a positive growth. Five replicates were performed.

### 2.4. Determination of the Minimum Biocidal Concentration

Each positive well was diluted from 10^−1^ to 10^−8^ in a new microtiter plate with NaCl 0.85%. Then, 10 μL of each well were plated in CASO following the methodology described by Drazic et al. (2015) [23]. The UFC were counted to determine the MBC.

### 2.5. Stress Conditions

Six bacterial pellets were resuspended in NaCl 0.85% to a final O.D = 2.5. Two bacterial suspensions were exposed to concentrations of sodium hypochlorite of 3.5 and 7 mgL^−1^, respectively, for 10 min. The other pair was exposed to concentrations of sodium hypochlorite of 3.5 and 7 mgL^−1^, respectively, for 30 min. Finally, the last two were used as the control and incubated for 10 and 30 min (without NaOCl).

Mineral media were added to the bacterial suspensions after each exposure time to a final concentration of minimal media (glucose 0.2 gL^−1^; NaH_2_PO_4_ 6 gL^−1^, KH_2_PO_4_ 3 gL^−1^, NH_4_Cl 1 gL^−1^, NaCl 0.5 gL^−1^, CaCl_2_ 0.012 gL^−1^, and MgSO_4_ 0.12 gL^−1^). We evaluated the effect of the disinfectant on the growth curve of *K. pneumoniae* by measuring the OD_600_ of the culture after the exposure to NaOCl. Samples also were taken to measure the production of metabolites by ^1^H-NMR.

### 2.6. Exposure to Sub-Lethal Concentration of Sodium Hypochlorite

A total of 100 mL of a sub-lethal concentration of sodium hypochlorite (7 mg L^−1^) was recirculated through the pipe containing the biofilm (0.5 mL min^−1^) for 10 min at 37 °C. The pellets of planktonic cells were resuspended in a solution of sodium hypochlorite (7 mg L^−1^) and NaCl (0.85%) and incubated for 10 min at 37 °C. The OD_600_ was equal to 2.5. Three of the bottles of biofilm and planktonic cells were used as controls, and no disinfectant was added. The metabolism was reactivated in the presence of the disinfectant, adding minimal media to the biofilm-recirculation and the planktonic-cell bottles. Then, they were incubated at 37 °C for 1 h. Two samples of 5 mL of each bottle were quenched with liquid nitrogen.

### 2.7. ^1^H-NMR Metabolic Profiling and Data Analysis

The samples were thawed over ice to measure the metabolites. Aliquots of 600 uL were centrifuged at 16,900 RCF and 4 °C for 5 min to separate the cell debris. A mixture of 1:10 of phosphate-TSP buffer (1.5 M KH_2_PO_4_, pH 7.4, 0.1% TSP, D_2_O) and the samples were prepared. The ^1^H-NMR spectrum was acquired using a spectrometer (Bruker Avance II Ultrashield 400 MHz (Bruker BioSpin, Rheinstetten, Germany)). Two measurements of each sample were performed at 300 K, for a total of 6 measurements per group. The standard 1D noesygppr1d pulse sequence was used, using a relaxation delay of 3 s, 64 scans, and an acquisition time of 4 s. Initially, the spectra phase and baseline correction were performed automatically using MestRenova v. 12.0.0 (Mestrelab Research S.L). The signals of the metabolites were deconvoluted using qGSD. All the spectral data were pre-processed using the R software package *metabom8* v. 0.4.4 (T Kimhofer). The area under the curve was measured to quantify the metabolites using the signal of the TSP as an internal standard.

### 2.8. Putative Metabolite Identification and Metabolic Pathways and Statistical Analysis

Metabolites were putatively identified using the software Chenomx and contrasting the chemical shift and coupling constant of the signals with the NMR spectra available in the Escherichia coli Metabolome Database (ECMDB). The mean and standard deviation of the concentration of the metabolites for the control and treatment replicates was determined. The normality of the data and the equality of variances between the groups were evaluated using the Shapiro–Wilk and Levene tests, respectively. Finally, the means between the groups were compared with a *t*-test.

## 3. Results

During their growth in minimal media, five metabolites were identified in biofilm and planktonic cultures (ethanol: ^1^H-NMR (400 MHz) [δ_H_ 1.18 (3H, t, J = 7.11 Hz), 3.67 (2H, q, J = 7.06)]; lactate [δ_H_ 1.33 (3H, d, J = 6.81 Hz), 3.67 (2H, q, J = 7.06)]; acetate [δ_H_ 1.92 (3H, s)]; succinate [δ_H_ 2.40 (4H, s)]; and formate [δ_H_ 8.46 (1H, s)]).

### 3.1. Determination of Sub-Lethal Concentration of Sodium Hypochlorite Disinfection and MBC

**Figure 2:**
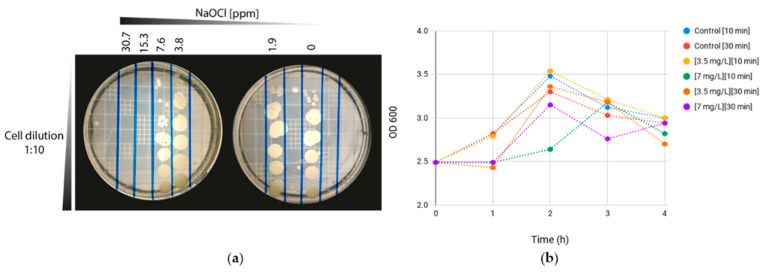
Determination of stress conditions. (**a**) MBC determination by microdilution/plating. (**b**) *K. pneumoniae* growth curves in the minimal mineral medium after stress with sodium hypochlorite. Controls correspond to the bacterial suspension without NaOCl addition but incubated under the same conditions as the treatments. The concentration of sodium hypochlorite used, and the contact time, are indicated in square brackets.

In its planktonic state, *K. pneumoniae* cells were killed after 10 min of exposure to concentrations above 7 mg L^−1^ of sodium hypochlorite (Figure 2a). This concentration was then used to evaluate the effect of the disinfectant on the growth of *K. pneumoniae* by measuring the OD_600_ of the culture after the exposure to NaOCl and reactivation of the metabolism by adding minimal media (Figure 2b, Table S1).

**Figure 3:**
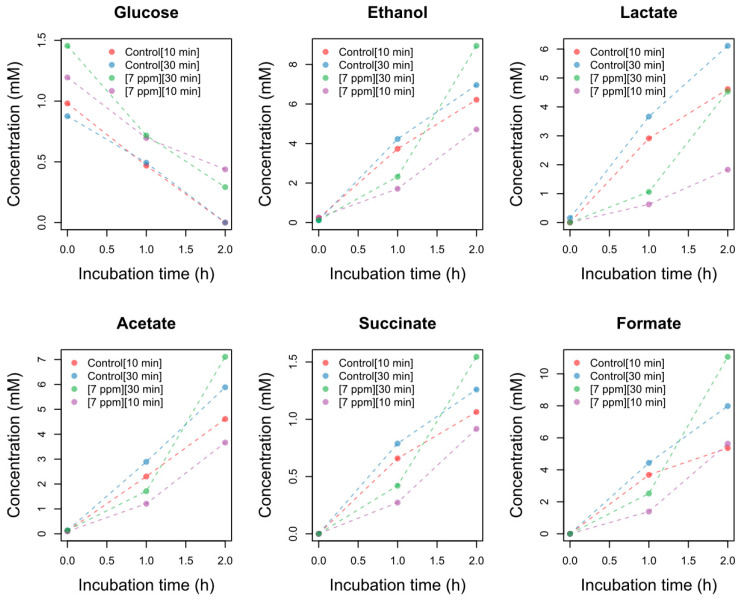
Intake of glucose and production of different metabolites in the minimal mineral medium after recovery from stress with sodium hypochlorite. The quantification of the metabolites was obtained from different ^1^H NMR spectra. The concentration of sodium hypochlorite used, and the exposure time, are indicated in square brackets.

The growth curves showed that there was, as expected, a decrease in the growth rate after exposure to the disinfectant. However, the growth curves are slightly different, showing a higher decrease in the growth rate when using a concentration of 7 mgL^−1^ for 10 min. With this disinfectant concentration, there were no changes in the OD of the culture after 1 h of incubation, but the cells were metabolically active, consuming the glucose in the minimal media and producing the metabolites mentioned previously (Figure 3, Table S2).

### 3.2. Concentration of Total and Free Chlorine during Stress

**Figure 4:**
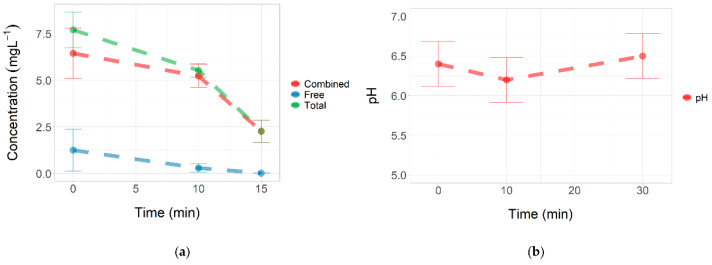
Physicochemical parameters. (**a**) Total and free chlorine concentrations during stress. (**b**) pH. The vertical bars show the maximum and minimum values found for three independent replicates.

The total and free chlorine levels decreased throughout the treatment. After 10 min of incubation, we found about 5.5 mg L^−1^ (±0.4) of total chlorine and 0.3 mg L^−1^ (±0.2) of free chlorine. The addition of minimal media to the bacterial suspension decreased the concentration of total chlorine and free chlorine to 2.3 mg L^−1^ (±0.6) and less than 0.02 mg L^−1^, respectively (Figure 4a, Table S3).

On the other hand, the pH of the bacterial suspension was between 6.0 and 6.6 (Figure 4b, Table S4). In these acidic pH conditions, the formation of HOCl is favored, which is the chlorine species that shows the highest bactericidal effect.

### 3.3. Metabolic Changes in Planktonic Cells and Biofilms

**Figure 5:**
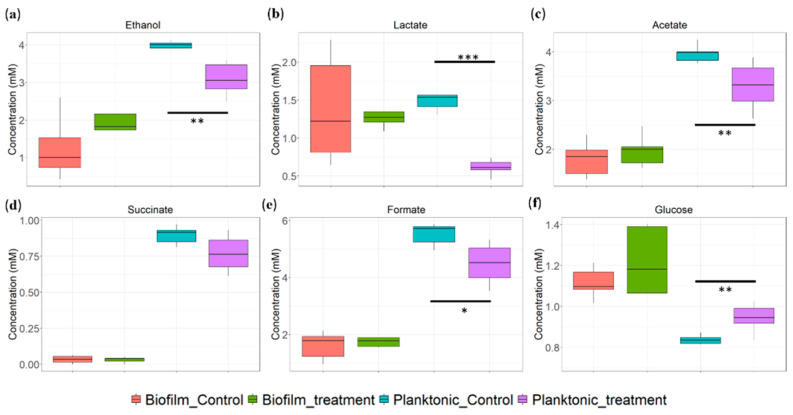
Effect of the sublethal concentration of sodium hypochlorite on the metabolic profile. (**a**) Ethanol. (**b**) Lactate. (**c**) Acetate. (**d**) Succinate. (**e**) Formate. (**f**) Glucose. The number of * represents the level of significance (* *p*-value < 0.05, ** *p*-value < 0.005, *** *p*-value < 0.0005).

Statistical analysis of the data showed a significant decrease in glucose intake in the planktonic cells after treatment with a sublethal concentration of NaOCl. Consequently, the production of upstream metabolites, such as ethanol, lactate, acetate, and formate, decreased significantly (Figure 5, Table S5). In contrast, the heterogeneity in the metabolic behavior of the biofilms was higher, and there was no significant effect of the treatment. The concentrations of succinate were close to the LOD (Table 1). The results of the normality tests, homogeneity of variances, and t-test are summarized in Tables S6–S8. 

## 4. Discussion

Sublethal doses of NaOCl were used to keep the planktonic and biofilm cells alive and evaluate the damage produced by the disinfectant. The results suggest that the damage occurs initially in the carbohydrate transport process. In the planktonic cells, the disruption of glucose intake significantly decreased the production of upstream metabolites such as ethanol, lactate, and acetate, while in biofilms, there were no changes in the metabolism (Figure 5). Structural changes in the capsule material are likely to have occurred in both cases but were not detected. Indeed, in experiments with different disinfectants with a similar oxidation power, we observed changes in the morphology of the capsules of *K. pneumoniae* [21].

### 4.1. Planktonic Cells Damage

The pH levels during disinfection were between 6.0 and 6.6. This condition favored the presence of free chlorine in the form of HOCl. According to Fukuzaki’s model [24], HOCl shows a higher bactericidal effect due to its neutral electric charge (compared to ClO-) and low molecular weight.

The HOCl could penetrate the cell membrane by passive diffusion, producing a double attack. First, by oxidizing the external components of the bacteria surface, such as the capsule, the cell wall, and cell membrane; and second, by oxidizing the internal components of the bacterial cell that are highly nucleophilic.

**Figure 6:**
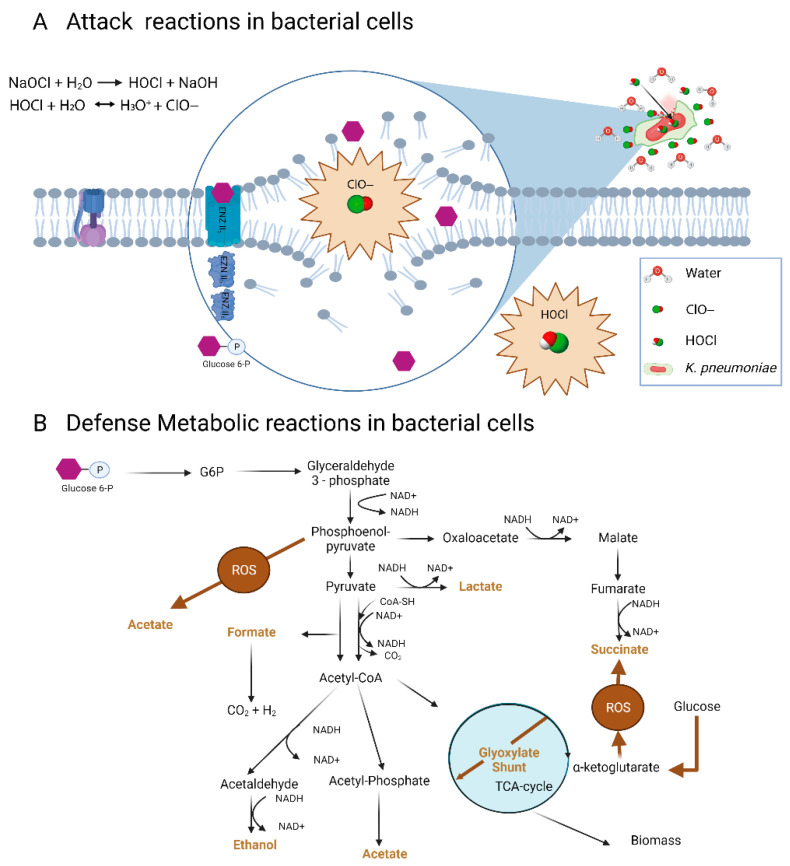
Proposed mechanism of action of sodium hypochlorite on *K. pneumoniae* cells. (**A**) Glucose transport disruption. (**B**) Changes in the metabolic pathways in response to oxidative stress. Image created with BioRender.com.

The *K. pneumoniae* capsule shows an anionic nature [25], which can promote electrostatic repulsion of the negatively-charged hypochlorite ions. The ClO- probably induces a rupture and disintegration of the capsule, wall, and cell membrane, inactivating glucose phosphorylation that occurs via group translocation of sugars with the participation of enzymes embedded in the cell membrane (Figure 6A). The non-phosphorylated glucose can accumulate in the cell or remain in the environment. This effect was observed previously in oxidative stress experiments with photocatalysis and TiO_2_ in *E. coli* for multiple sugars, including glucose [26].

In Figure 6B, the central metabolism of the glucose of *K. pneumoniae* can be observed, as described by Lu et al. [27]. During exposure to sub-lethal concentrations of NaOCl, the repairing mechanisms are activated, disrupting cell respiration. Prokaryotes have developed antioxidant defense systems through scavenging enzymes, such as superoxide dismutase (SOD), peroxiredoxin, and catalase, to protect cells from ROS damage [28,29]. Apart from antioxidant defense systems, metabolism remodeling plays a crucial role in mitigating oxidative damages [30]. In biofilms and planktonic cells, metabolites derived from the TCA oxidative metabolism and fermentation pathways were observed. Since the intake of glucose is disrupted, the activity of these upstream pathways decreases, affecting the production of metabolites such as formate, ethanol, acetate, lactate, and succinate (Figure 6B). Other studies in *E. coli* have shown that oxidative stress results in a sub-regulation of central carbon pathways, such as glycolysis and the Krebs cycle, where glucose flow is redirected to the NADPH-production pathways such as pentose phosphate, which plays a fundamental role in the metabolic response to oxidative stress [30]. It also has been proposed that low levels of formate and lactic acid may reflect a metabolic shift toward pyruvate and Acetyl-CoA production [23]. In Figure 2, it was observed that the total and free chlorine concentrations gradually declined over time; consequently, the metabolic behavior might reflect the changes in the biocidal mechanism of hypochlorite throughout its interaction with the bacteria.

### 4.2. Biofilm Defense Strategy

**Figure 7:**
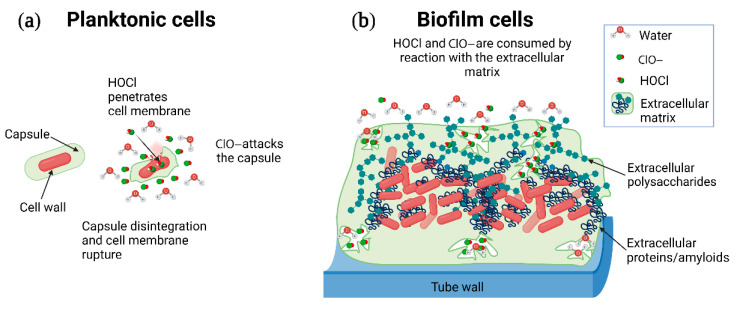
Protective role of the biofilm matrix against chlorine disinfection. (**a**) Planktonic cells have a larger surface area exposed to the attack of HOCl and ClO-^−^. (**b**) A more complex and thicker extracellular biofilm matrix increases the chlorine demand as the HOCl and ClO- are consumed by reaction with the extracellular matrix.

The defensive barriers of biofilms have been extensively researched for different groups of bacteria, and extensive work has been done with *P. aeruginosa* and *S. aureus*. In these previous works, it has been argued that this defense could be more chemical than physical. It is well known that the extracellular matrix surrounding the biofilms acts as a natural barrier that protects the cell from disinfectants and antibiotics by reacting with them and limiting its diffusion inside the cell [31,32]. The biofilm also can decrease the bacterial surface area exposed to the attack of the disinfectant. These defense mechanisms cause a sharp increase in the concentration of sodium hypochlorite required to eliminate the biofilms (Figure 7).

However, it has been suggested that biofilms are protected by a mechanism other than superficial physical shielding by the biofilm matrix because a low bactericidal effect is observed despite direct detection of efficient physical penetration of the antimicrobial agent [33]. Our results showed no changes in the biofilm metabolic footprint in response to the disinfection under the evaluated conditions. However, future studies are required to evaluate whether the response of biofilms to higher concentrations of sodium hypochlorite is the same as that observed in planktonic cells.

More research is needed on the killing effect of disinfectant agents on biofilms. Most studies focus on planktonic cells, but biofilms and bacterial aggregates are commonly found in water environments. This work demonstrates the versatility of NMR in monitoring the metabolic response of biofilms and planktonic cells to disinfection. The quantification of multiple metabolites can be performed easily compared to separation techniques such as HPLC/MS. Multiple, distinct NMR resonances are observed per molecule, eliminating the need for chromatographic separation and increasing metabolite identification accuracy.

## Figures and Tables

**Table 1:** Concentration of metabolites produced by *K. pneumoniae* biofilm cells and planktonic cells with and without exposure to sodium hypochlorite.

Metabolite (mM) (±Standard Deviation)	Biofilm Control (without NaOCl)	Biofilm Treatment (NaOCl)	Planktonic Control (without NaOCl)	Planktonic Treatment (NaOCl)
Ethanol	1.23 (0.79)	2.15 (1.14)	3.94 (0.21)	3.09 (0.44)
Lactate	1.38 (0.72)	1.29 (0.17)	1.48 (0.11)	0.61 (0.09)
Acetate	1.80 (0.35)	1.97 (0.33)	3.91 (0.25)	3.30 (0.48)
Succinate	0.03 (0.03)	0.03 (0.02)	0.89 (0.06)	0.77 (0.12)
Formate	1.62 (0.49)	1.86 (0.37)	5.52 (0.39)	4.49 (0.71)
Glucose	1.12 (0.07)	1.22 (0.17)	0.82 (0.05)	0.94 (0.07)

## Data Availability

Not applicable.

## Supplementary Materials

The following supporting information can be downloaded at: https://www.mdpi.com/article/10.3390/microorganisms10071323/s1, Table S1. Growth of *K. pneumoniae* measured by OD after the exposure to NaOCl and reactivation of the metabolism by adding minimal media, Table S2. Intake of glucose and production of different metabolites in the minimal mineral medium after recovery from stress with sodium hypochlorite, Table S3. Total and free chlorine concentrations during stress, Table S4. pH during stress, Table S5. Effect of the sublethal concentration of sodium hypochlorite on the metabolic profile, Table S6. Shapiro-Wilk normality test, Table S7. Levene’s test for homogeneity of variances, Table S8. *t*-test of means differences.
