# Supplementary material for: Analysis of the Metabolic Response of Planktonic Cells and Biofilms of Klebsiella pneumoniae to Sublethal Disinfection with Sodium Hypochlorite Measured by NMR
Source: Microorganisms. 2022 Jun 30;10(7):1323. doi: 10.3390/microorganisms10071323 (PMC9323045; doi:10.3390/microorganisms10071323)
Supplement: Supplementary file 1 [file microorganisms-10-01323-s001.zip › microorganisms-1778760-supplementary.pdf]

Supplementary material

Analysis of the Metabolic Response of Planktonic Cells and Biofilms of *Klebsiella pneumoniae* to Sublethal Disinfection with Sodium Hypochlorite Measured by NMR

David Felipe Garcia Mendez [1,2], Julián Andrés Rengifo Herrera [3], Janeth Sanabria [2,4,*] and Julien Wist [1,2]

[1] Chemistry Department, Universidad del Valle–Sede Meléndez, Calle 13 # 100-00 Cali, Colombia; david.garcia.mendez@correounivalle.edu.co (D.F.G.M.); julien.wist@murdoch.edu.au (J.W.)
[2] Australian National Phenome Center, Murdoch University, Perth, WA 6150, Australia
[3] Centro de Investigación y Desarrollo en Ciencias Aplicadas "Dr. J.J. Ronco" (CINDECA), Departamento de Química, Facultad de Ciencias Exactas, UNLP-CCT La Plata, CONICET, 47 No. 257, La Plata 1900, Argentina; julianregifo@quimica.unlp.edu.ar
[4] Environmental Microbiology and Biotechnology Laboratory, Engineering Faculty, Engineering School of Environmental & Natural Resources, Universidad del Valle–Meléndez campus, Cali, Colombia
* Correspondence: janeth.sanabria@correounivalle.edu.co

Table S1. Growth of *K. pneumoniae* measured by OD after the exposure to NaOCl and reactivation of the metabolism by adding minimal media

	OD_{600}					
Time (h)	Control [10 min]	Control [30 min]	[3.5 mgL^{-1}] [10 min]	[7 mgL^{-1}] [10 min]	[3.5 mgL^{-1}] [30 min]	[7 mgL^{-1}] [30 min]
0	2.49	2.49	2.49	2.49	2.49	2.49
1	2.82	2.82	2.79	2.49	2.43	2.49
2	3.48	3.3	3.54	2.64	3.36	3.15
3	3.12	3.03	3.21	3.18	3.18	2.76
4	3	2.94	3	2.82	2.7	2.94

Table S2. Intake of glucose and production of different metabolites in the minimal mineral medium after recovery from stress with sodium hypochlorite.

	Metabolite concentration (mM)					
	Ethanol	Lactate	Acetate	Succinate	Formate	Glucose
Control[10 min]	0.22	0	0.11	0	0	0.98
Control[30 min]	4.23	3.66	2.89	0.79	4.43	0.49
[7 ppm][10 min][1 h]	1.72	0.63	1.21	0.27	1.39	0.70
[7 ppm][30 min][2 h]	8.94	4.53	7.11	1.54	11.06	0.29
[7 ppm][10 min][0 h]	0.26	0	0.10	0	0	1.19
Control[10 min][2h]	6.22	4.61	4.61	1.06	5.36	0
[7 ppm][30 min][1 h]	2.32	1.06	1.71	0.42	2.52	0.72
Control[10 min][1 h]	3.73	2.91	2.30	0.66	3.68	0.47
[7 ppm][30 min][0 h]	0.12	0	0.14	0	0	1.45
Control[30 min][2 h]	6.96	6.11	5.89	1.26	7.99	0
[7 ppm][10 min][2 h]	4.71	1.83	3.67	0.92	5.64	0.44
Contro[30 min][0 h]	0.11	0.16	0.13	0	0	0.88

Table S3. Total and free chlorine concentrations during stress.

Type	Time (min)	Concentration (mgL^{-1})
Total chlorine	0	8.6
Total chlorine	0	7.8
Total chlorine	0	6.7
Total chlorine	10	5.7
Total chlorine	10	5.8
Total chlorine	10	5.1
Total chlorine	15	2.9
Total chlorine	15	2.2
Total chlorine	15	1.7
Free chlorine	0	0.7
Free chlorine	0	2.55
Free chlorine	0	0.5
Free chlorine	10	0.25
Free chlorine	10	0.1
Free chlorine	10	0.55
Free chlorine	15	0.02
Free chlorine	15	0.02
Free chlorine	15	0.02
combined chlorine	0	7.9
combined chlorine	0	5.25
combined chlorine	0	6.2
combined chlorine	10	5.45
combined chlorine	10	5.7
combined chlorine	10	4.55
combined chlorine	15	2.9
combined chlorine	15	2.2
combined chlorine	15	1.7

Table S4. pH during stress

Type	Time	Concentration
pH	0	6.6
pH	0	6.2
pH	10	6.4
pH	10	6.0
pH	30	6.3
pH	30	6.7

Table S5. Effect of the sublethal concentration of sodium hypochlorite on the metabolic profile.

Cell type	Group	Ethanol	Lactate	Acetate	Succinate	Formate	Glucose
Biofilm_control	Control	1.66	0.68	1.40	0.01	1.05	1.09
Biofilm_control	Control	0.43	0.64	1.39	0.00	0.96	1.08
Biofilm_control	Control	1.15	1.25	2.29	0.06	2.14	1.19
Biofilm_control	Control	0.70	2.19	1.81	0.03	1.77	1.02
Biofilm_control	Control	0.86	2.30	1.88	0.04	1.98	1.10
Biofilm_control	Control	2.60	1.19	2.02	0.06	1.80	1.21
Biofilm_treatment	Treat	1.82	1.09	2.05	0.04	1.89	1.06
Biofilm_treatment	Treat	4.04	1.35	1.72	0.00	1.58	1.40
Biofilm_treatment	Treat	1.74	1.55	2.47	0.05	2.48	1.07
Biofilm_treatment	Treat	0.98	1.27	1.62	0.02	1.56	1.18
Biofilm_treatment	Treat	2.16	1.21	2.00	0.04	1.78	1.39
Planktonic_treatment	Treat	2.77	0.74	2.96	0.68	3.90	1.00
Planktonic_treatment	Treat	3.60	0.64	3.71	0.85	4.80	0.92
Planktonic_treatment	Treat	2.50	0.59	2.63	0.61	3.53	0.92
Planktonic_treatment	Treat	3.01	0.46	3.08	0.68	4.24	1.03
Planktonic_treatment	Treat	3.59	0.69	3.88	0.93	5.33	0.97
Planktonic_treatment	Treat	3.11	0.58	3.56	0.87	5.11	0.84
Planktonic_Control	Control	3.99	1.57	4.00	0.91	5.79	0.85
Planktonic_Control	Control	3.89	1.38	3.77	0.83	5.10	0.87
Planktonic_Control	Control	3.53	1.31	3.49	0.81	4.96	0.82
Planktonic_Control	Control	4.04	1.52	4.25	0.97	5.86	0.72
Planktonic_Control	Control	4.14	1.56	4.01	0.93	5.77	0.83
Planktonic_Control	Control	4.06	1.57	3.98	0.92	5.68	0.84

Table S6. Shapiro-Wilk normality test.

Planktonic cells				
	Control		Treatment	
Metabolite	W	p-value	W	p-value
Ethanol	0.89137	0.1104	0.92336	0.5299
Lactate	0.80267	0.06211	0.96821	0.8802
Acetate	0.93012	0.5811	0.94313	0.6845
Succinate	0.93373	0.3964	0.89304	0.3344
Formate	0.79778	0.05612	0.94826	0.7262
Glucose	0.82885	0.1051	0.96081	0.826
Biofilm cells				
	Control		Treatment	
Metabolite	W	p-value	W	p-value
Ethanol	0.91529	0.4721	0.87488	0.2868
Lactate	0.8635	0.2015	0.9752	0.9074
Acetate	0.92652	0.5532	0.94226	0.6819
Succinate	0.93164	0.5928	0.93013	0.5972
Formate	0.85848	0.184	0.84372	0.1755
Glucose	0.93358	0.608	0.82118	0.1192

Table S7. Levene's test for homogeneity of variances.

Planktonic cells				
Metabolite	F	Num df	Denom df	p-value
Ethanol	0.24319	5	5	0.1469
Lactate	1.3	5	5	0.7804
Acetate	0.28226	5	5	0.1913
Succinate	0.22645	5	5	0.1288
Formate	0.30412	5	5	0.2174
Glucose	0.64594	5	5	0.6432
Biofilm cells				
Metabolite	F	Num df	Denom df	p-value
Ethanol	0.47984	5	4	0.4411
Lactate	17.157	5	4	0.0166
Acetate	1.1294	5	4	0.9322
Succinate	1.5751	5	4	0.6805
Formate	1.7276	5	4	0.6164
Glucose	0.1927	5	4	0.1001

Table S8. T-test of means differences.

Planktonic cells			
Metabolite	t	df	p-value
Ethanol	4.2075	10	0.001807
Lactate	14.216	10	5.849E-08
Acetate	2.7095	10	0.02195
Succinate	2.1824	10	0.05403
Formate	3.1467	10	0.01039
Glucose	-3.5128	10	0.005605
Biofilm cells			
Metabolite	t	df	p-value
Ethanol	-1.5706	9	0.1507
Lactate	0.24471	9	0.8122
Acetate	-0.82539	9	0.4305
Succinate	0.17895	9	0.8619
Formate	-0.8905	9	0.3964
Glucose	-0.13892	9	0.1982
